# Performance comparison of ultrasonography and magnetic resonance imaging in their diagnostic accuracy of placenta accreta spectrum disorders: a systematic review and meta-analysis

**DOI:** 10.1186/s13244-022-01192-w

**Published:** 2022-03-22

**Authors:** Shibin Hong, Yiping Le, Ka U. Lio, Ting Zhang, Yu Zhang, Ning Zhang

**Affiliations:** 1 Department of Obstetrics and Gynecology, Ren Ji Hospital, Shanghai Jiao Tong University School of Medicine, 160 Pujian Road, Pudong New District, Shanghai, 200127 China; 2 Shanghai Key Laboratory of Gynecologic Oncology, Ren Ji Hospital, Shanghai Jiao Tong University School of Medicine, 160 Pujian Road, Shanghai, 200127 China; 3 Department of Medicine, Temple University Hospital, Lewis Katz School of Medicine at Temple University, 3401 N Broad St, Philadelphia, PA 19140 USA; 4 Department of Obstetrics and Gynecology, Hainan Women and Children’s Medical Center, Changbin Road, Haikou City, 570312 Hainan Province China

**Keywords:** Ultrasonography, Magnetic resonance imaging, Placenta accrete spectrum disorders, Meta-analysis

## Abstract

**Objectives:**

Accurate prenatal diagnosis of placenta accrete spectrum disorder (PAS) remains a challenge, and the reported diagnostic value of ultrasonography (US) and magnetic resonance imaging (MRI) varies widely. This study aims to systematically evaluate the diagnostic accuracy of US as compared with MRI in the detection of PAS within the identical patient population.

**Methods:**

Medline, EMBASE, Google scholar and Cochrane library were searched. Pooled sensitivity, specificity, diagnostic odds ratio (DOR) and the area under the summary receiver operating characteristic (SROC) curve were calculated. Subgroup analysis was also performed to elucidate the heterogeneity of results.

**Results:**

A total of 18 articles comprising 861 pregnancies were included in the study. The overall diagnostic accuracy of US for identification of PAS was as follows: sensitivity [0.90 (0.86–0.93)], specificity [0.83 (0.79–0.86)], DOR [39.5 (19.6–79.7)]. The overall diagnostic accuracy of MRI for identification of PAS was as follows: sensitivity [0.89 (0.85-0.92)], specificity [0.87 (0.83–0.89)], DOR [37.4 (17.0–82.3)]. The pooled sensitivity (*p* = 0.808) and specificity (*p* = 0.413) between US and MRI are not significantly different. SROC analysis revealed that there was no statistical difference (*p* = 0.552) in US and MRI for the overall predictive accuracy of PAS. Furthermore, in the subgroup analysis of between retrospective and prospective studies, between earlier and most recent studies, there was no statistical difference (*p* > 0.05) in diagnostic accuracy of US and MRI for the detection of PAS.

**Conclusions:**

Both ultrasonography (US) and magnetic resonance imaging (MRI) showed comparable accuracy in the prenatal diagnosis of placenta accrete spectrum disorder (PAS). Routine employment of MRI with relatively high expense in the prenatal identification of PAS should not be recommended.

## Key points

- Both US and MRI showed comparable accuracy in the prenatal diagnosis of PAS.
- MRI should not be used routinely as an adjunct tool to US in the diagnosis of PAS.
- The comprehensive scoring system using US, MRI and clinical risk factors can help to improve accurate prenatal diagnosis of PAS.

## Introduction

Placenta accrete spectrum (PAS) disorder is a growing obstetric concern. The rise in cesarean section rates worldwide over the past decades has led to a fundamental increase in the prevalence of PAS [1]. PAS can be classified into three major variants of placenta accreta, placenta increta and placenta percreta according to the degree of trophoblastic invasion through the myometrium and the uterine serosa [2].

Various predisposing factors have been identified for the development of PAS; this includes placenta previa, prior cesarean delivery, multiparity, advanced maternal age (> 35 years), myometrial scaring from prior uterine surgery, and assisted reproductive techniques. Of which, the combination of placenta previa and previous cesarean delivery is the most commonly reported risk factor. PAS leads to the formation of hypertrophied, disorganized uteroplacental vascularity at the implantation site, and the immediate clinical consequence is massive hemorrhage while removing the placenta manually after delivery. PAS is the most common indication for emergent peripartum hysterectomy [3, 4] and also leads to extensive morbidities (i.e., massive transfusion, disseminated intravascular coagulation, infection, sepsis) and surgical-related complications (i.e., pelvic organs injury, fistula formation) [5].

Prenatal diagnosis of PAS allows for meticulous planning and multidisciplinary management of patients, thus substantially improve the maternal prognosis. However, accurate identification of morbidly adherent placenta before delivery remains a challenge, and almost half to two-thirds of PAS remain undiagnosed before delivery indicated by recent studies [6, 7]. Ultrasonography (US) is the most commonly used diagnostic modality for detecting abnormal placentation, while magnetic resonance imaging (MRI) is usually recommended as an adjunctive diagnostic modality and reserved for cases with inconclusive ultrasound findings. Although the diagnostic values of US and MRI has been previously studied, there is a high variability in the reported diagnostic accuracy, which can be attributed by the relative rarity of the disease and the subjectivity of the imaging modalities largely dependence on the experiences of the sonographer or radiologist.

Therefore, this study was performed to evaluate and compare the diagnostic performance of the two imaging modalities (US and MRI) for the prenatal identification of PAS. To our knowledge, this study is the first systemic review and meta-analysis comparing the predictive accuracy between MRI and US in PAS within the identical patient population.

## Materials and methods

### Search strategy

A systemic literature search using Medline, Embase, Google Scholar, the Cochrane Database of Systematic Reviews (CDSR), the Cochrane Central Register of Controlled Trials (CENTRAL), and Database of Abstracts of Reviews of Effects (DARE) up to May 28, 2021, using the relevant keywords: “magnetic resonance imaging,” “ultrasound,” “sonography,” “ultrasonography,” “placenta accrete,” “invasive placenta,” “placenta increta,” as well as combinations of these terms. Two authors independently reviewed the abstracts and full-text; relevant data were collected and analyzed.

### Study selection

Two independent investigators reviewed the literature and performed the study selection. Inconsistencies were discussed among the reviewers, and consensus was reached.

Inclusion criteria for this meta-analysis were as follows:

1. Confirmation of PAS defined as histopathological evidence and/or intraoperative finding of gross placenta invasion.
2. Prospective cohorts and retrospective cohorts studies evaluating the predictive value of both US and MRI within the same patient population.
3. Presence of US signs indicative of PAS, using the reference findings as follows: prominent lacunae, myometrial thinning, focal exophytic masses, bladder line interruption, increased vascularity.
4. Presence of MRI signs indicative of PAS, using the reference findings as follows: intraplacental dark bands with a random pattern on T2 weighted sequences, heterogeneous signal intensity in the placenta, focal uterine bulge, focal interruptions of the uterine wall, and tenting of the bladder.

Exclusion criteria for this meta-analysis were as follows:

1. Opinions, editorials, review articles, case reports, and studies without the necessary variables mentioned above.
2. Absence of a precise number of participants, true positives (TP), false positives (FP), false negatives (FN), and true negatives (TN) of MRI and US assessment or such data cannot be calculated.

### Data extraction

Two authors independently collected data based on the inclusion criteria and exclusion criteria. The following information was obtained and listed from the included study: year of publication, first author’s name and country, number of patients, number of PAS, patient characteristics, study design (prospective or retrospective), technique aspects, image interpretation (blind or not). The absolute number of patients and relevant data including TP, FP, TN, and FN for US and MRI were collected from each included study. We calculated the above data if such information was not directly reported. We also contacted the authors for any missing data for the completion of data extraction.

### Quality Assessment

The updated QUADAS-2 (Quality Assessment of Diagnostic Accuracy Studies-2) criteria were employed to evaluate the risk of bias and applicability of the included studies. Each item was rated as “low,” “high,” or “unclear.” We scored the item as “unclear” when the absolute information was absent to conclude an accurate judgment.

### Statistical analysis

Meta-Disc software for meta-analysis was used for statistical analyses [8]. Statistical difference was defined as *p* < 0.05.

The heterogeneity of studies included was evaluated by *I*^2^ statistic and Cochran’s Q test. The study was defined as insignificant heterogeneity with *I*^2^ values of 0–25%, low heterogeneity with *I*^2^ values of 25–50%, moderate heterogeneity with *I*^2^ values of 50–75%, and high heterogeneity with *I*^2^ values of 75–100%. To calculate the overall diagnostic accuracy of US and MRI for the identification of PAS including pooled sensitivity and specificity, positive and negative likelihood ratio (LR+ and LR−), and diagnostic odds ratio (DOR), appropriate statistical modality was chosen according to the outcomes of heterogeneity test: a random effect model used when *I*^2^ exceeded 50%, a fixed-effect model used in other cases.

For comparison of diagnostic performance between US and MRI, the hierarchical summary receiver operating characteristics (SROC) curves were plotted. The area under the SROC curve (AUC) and the Q^*^ index (the point in SROC curve space closest to the ideal top-left corner and at which test sensitivity and specificity are equal) were also computed. To determine the difference of sensitivity, specificity, and AUS between US and MRI, Z value tests were performed. Additionally, sensitivity analysis was performed to assess the effect of each included study on the overall results.

## Results

### Literature search and general characteristics of the included studies

**Fig. 1:**
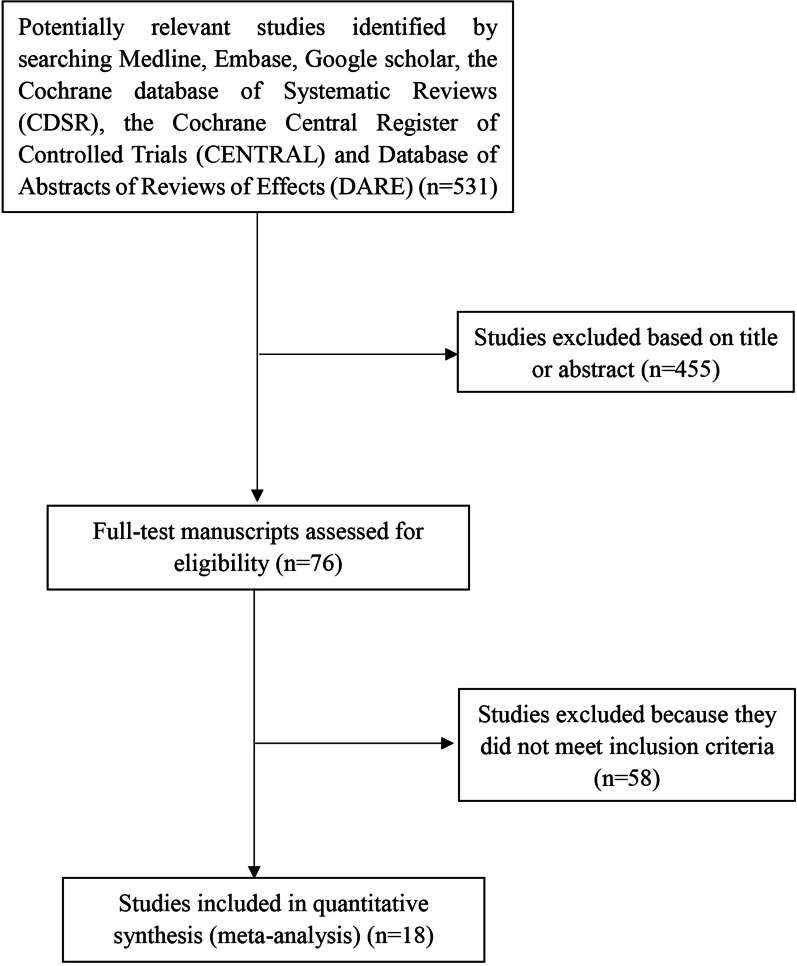
Flowchart of studies for meta-analysis

A total of 531 primary citations were identified from the comprehensive literature search. Among them, 455 studies were excluded based on screening the titles and abstracts. Two investigators carefully read the full-text manuscripts of the remaining 76 studies, and 58 studies were excluded according to the inclusion criteria. Finally, 18 studies (Levine [9], Masselli [10], Dwyer [11], Mansour [12], Lim [13], Elhawary [14], Peker [15], Riteau [16], Algebally [17], Satija [18], Balcacer [19], Rezk [20], Marcillac [21], Budorick [22], Kumar [23], Ayati [24], Romeo [25], Xia [26]) were included in our meta-analysis (Fig. 1). There is a total of 861 pregnancies in the included studies with a potential risk of PAS, and 339 (39.4%) pregnancies were diagnosed with PAS based on the histopathological evidence and/or intraoperative findings of gross placenta invasion. Of the included 18 studies, the majority of them (83.3%) specifically indicated that both the radiologists and sonographers reviewed and interpreted the images without prior knowledge of the pathological and surgical findings to avoid observer bias. The general characteristics of the included studies are summarized in Table 1.

**Table 1:** General characteristics of studies included in the systematic review (18 studies)

References	Country	Study design	Period analyzed	Trimester at scan	Reference standard	Mean age (years)	Image interpretation	Pregnancies	PAS at delivery
Levine [9]	USA	Prosp	1995–1997	2–3	Path/Surg	N/A	Blind	18	6
Masselli [10]	Italy	Prosp	2006–2007	3	Path/Surg	31	Blind & Experienced	50	12
Dwyer [11]	USA	Retro	2001–2006	N/A	Path/Surg	N/A	N/A	32	15
Mansour [12]	Egypt	Prosp	2010–2011	3	Surg	32	Blind & Experienced	35	15
Lim [13]	USA	Retro	2009–2010	2–3	Path	33	N/A	13	9
Elhawary [14]	Egypt	Retro	2010–2012	3	Surg	32	Blind	39	9
Peker [15]	Turkey	Retro	2008–2011	3	Path	N/A	Blind	40	20
Riteau [16]	France	Retro	2001–2012	3 (28–30)	Path/Surg	34	Blind & Experienced	42	26
Algebally [17]	Qatar	Prosp	2011–2014	3	NA	33	Blind & Experienced	100	32
Satija [18]	India	Prosp	2013–2014	3 (majority)	Path/Surg	29	Blind & Experienced	30	8
Balcacer [19]	USA	Retro	2004–2014	3 (majority)	Path/Surg	N/A	Blind & Experienced	40	18
Rezk [20]	Egypt	Prosp	2012–2014	3	Path/Surg	30	Blind & Experienced	74	53
Marcillac [21]	France	Retro	2010–2014	N/A	Path/Surg	32	Blind	22	13
Budorick [22]	USA	Retro	2006–2012	2–3	Path/Surg	34	Blind & Experienced	45	14
Kumar [23]	India	Prosp	2011–2013	2–3	Path/Surg	N/A	N/A	22	9
Ayati [24]	Iran	Prosp	2012–2013	2–3	Path/Surg	31	Blind & Experienced	82	17
Romeo [25]	Italy	Retro	2012–2018	3	Path/Surg	35	Blind & Experienced	51	23
Xia [26]	China	Retro	2012–2018	3	Path/Surg	36	Blind & Experienced	126	40

Only first author of each study is shown

PAS, placenta accrete spectrum disorder; Prosp, prospective; Retro, retrospective; Surg, surgical findings; Path, pathology; N/A, not available

**Fig. 2:**
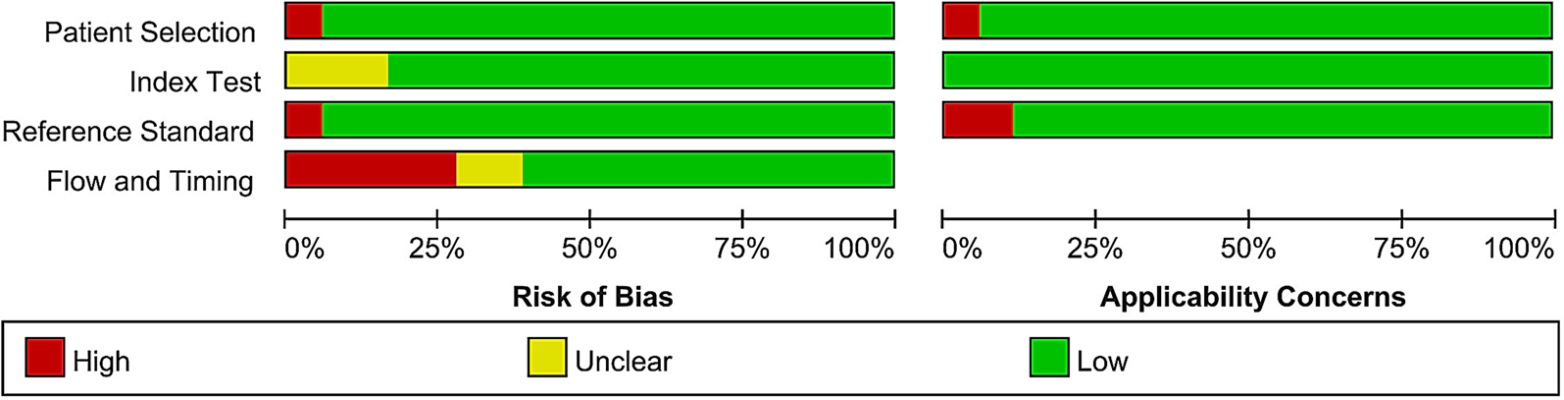
Summary results of QUADAS tool on risk of bias and applicability concerns for the included studies in the present systematic review

A quality assessment of the included 18 studies by the updated QUADAS-2 is outlined in Fig. 2. The results indicated an overall satisfactory quality of the identified studies. Furthermore, sensitivity analysis was conducted to assess the effect of each included study on the overall results by omitting one study at a time. No outlier was identified.

### Overall diagnostic accuracy and comparison between US and MRI for the prenatal identification of PAS

**Fig. 3:**
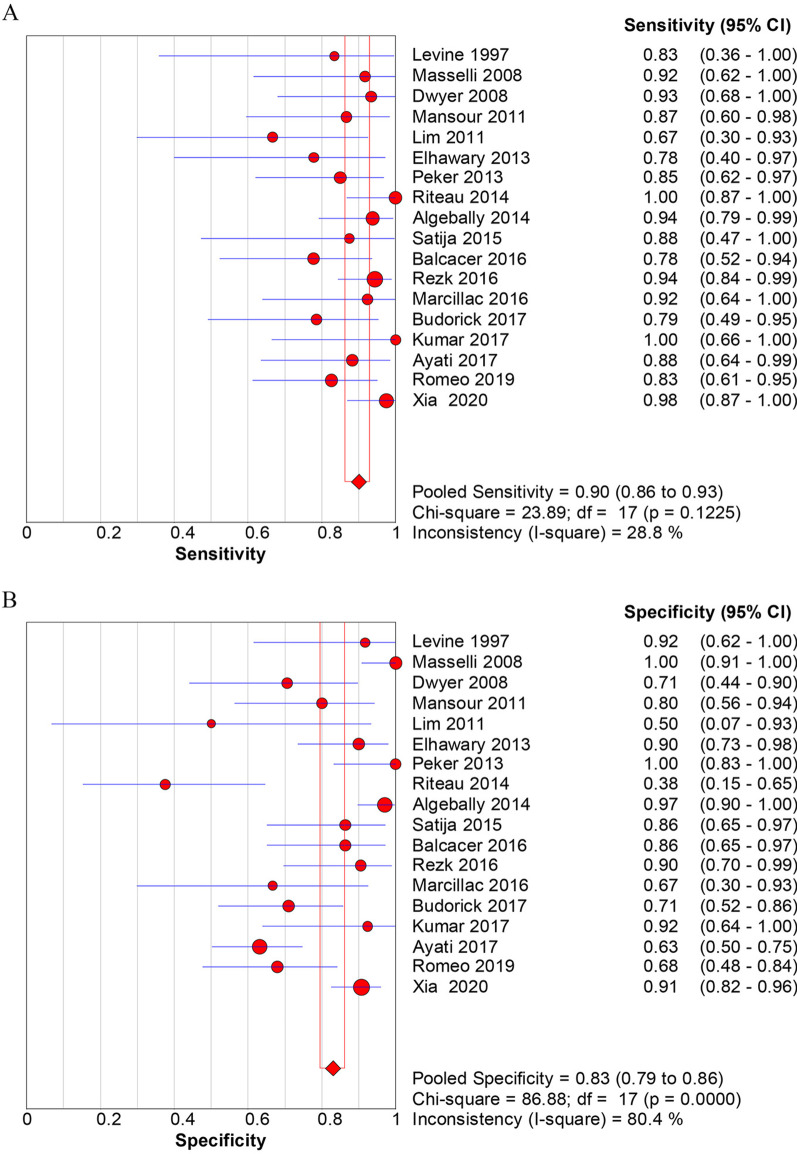
Forest plots of the overall sensitivity (**A**) and specificity (**B**) of diagnosis of placenta accrete spectrum disorder (PAS) with ultrasonography (US). The first author’s name of each study is listed. CI, confidence interval

**Fig. 4:**
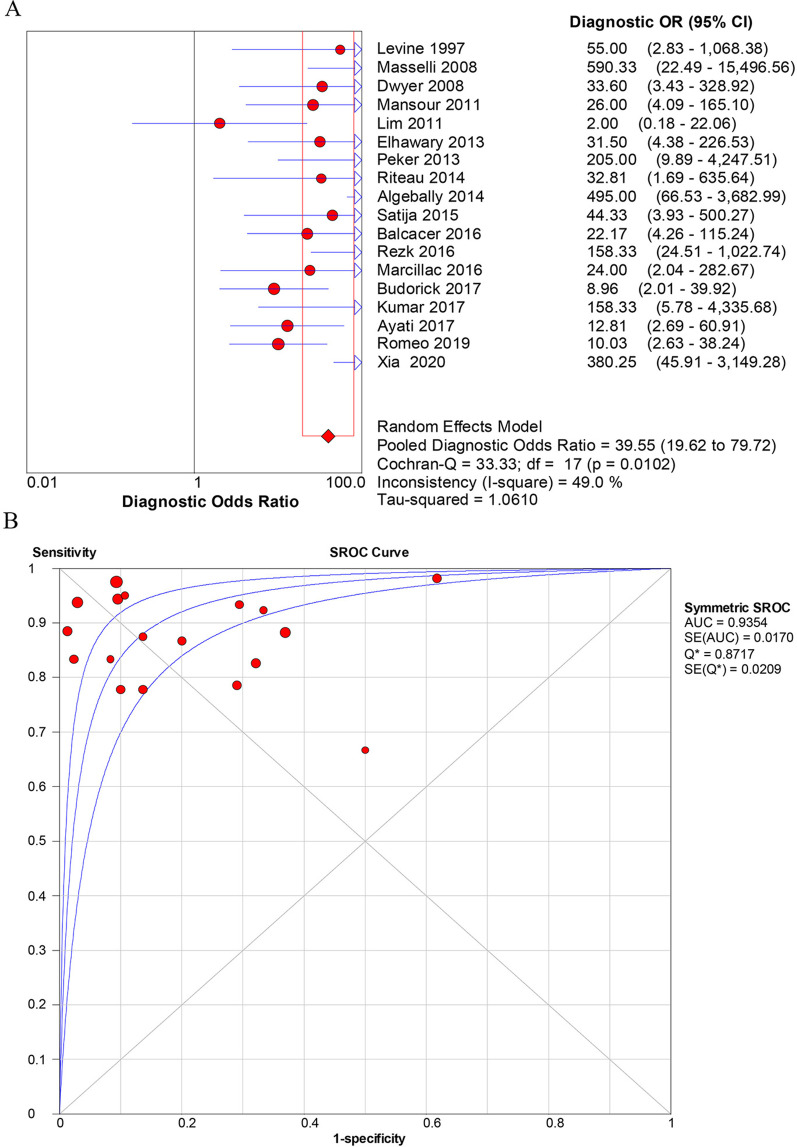
The diagnostic performance of ultrasonography (US) for the prenatal identification of placenta accrete spectrum disorder (PAS) based on the (**A**) forest plots of overall diagnostic odds ratio (DOR) and (**B**) summary receiver operating characteristic (SROC) curve. The weight of each study is reflected by the size of data points. AUC, area under the curve; SE, standard error

**Fig. 5:**
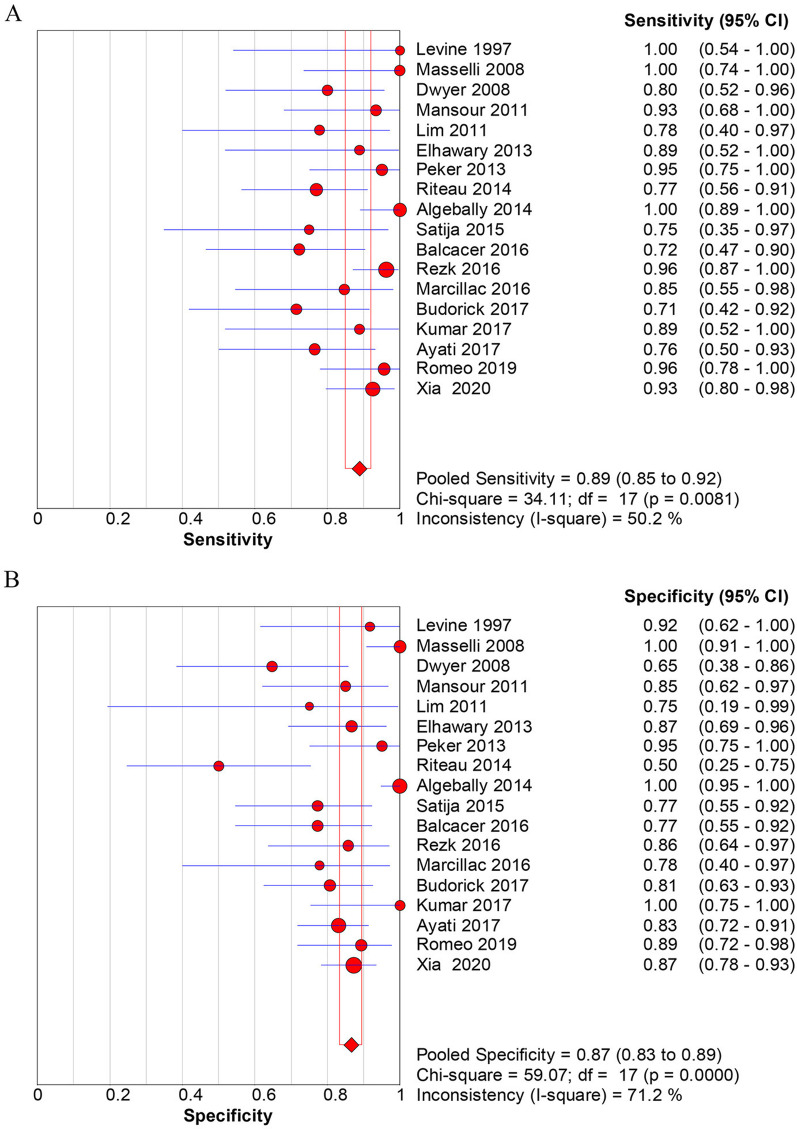
Forest plots of the overall sensitivity (**A**) and specificity (**B**) of diagnosis of placenta accrete spectrum disorder (PAS) with magnetic resonance imaging (MRI). The first author’s name of each study is listed. CI, confidence interval

**Fig. 6:**
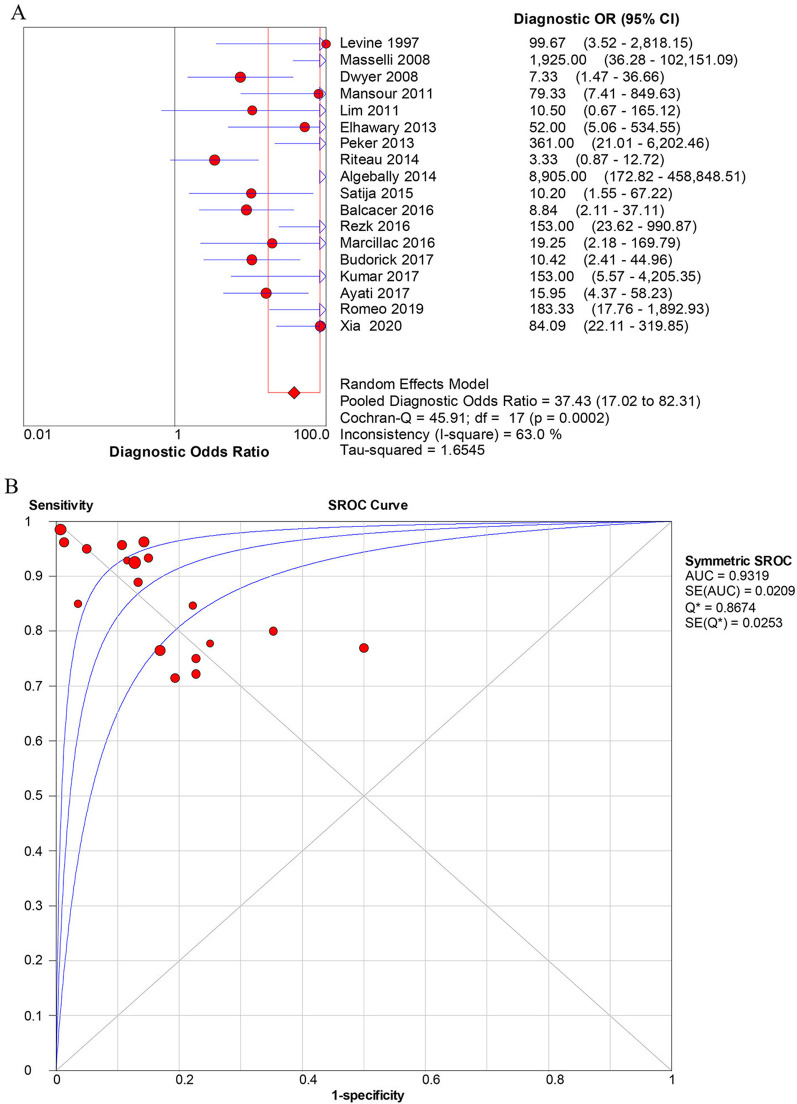
The diagnostic performance of magnetic resonance imaging (MRI) for the prenatal identification of placenta accrete spectrum disorder (PAS) based on the **A** forest plots of overall diagnostic odds ratio (DOR) and **B** summary receiver operating characteristic (SROC) curve. The weight of each study is reflected by the size of data points. AUC, area under the curve; SE, standard error

The overall diagnostic accuracy of US for identification of PAS was as follows: sensitivity [0.90 (0.86–0.93)] (Fig. 3A), specificity [0.83 (0.79–0.86)] (Fig. 3B), positive likelihood ratios (LR +) [4.85 (3.08–7.64)], negative likelihood ratios (LR-) [0.16 (0.12–0.23)], DOR [39.5 (19.6–79.7)] (Fig. 4A). The overall diagnostic accuracy of MRI for identification of PAS was as follows: sensitivity [0.89 (0.85–0.92)] (Fig. 5A), specificity [0.87 (0.83–0.89)] (Fig. 5B), LR+ [5.16 (3.42–7.78)], LR− [0.17 (0.11–0.27)], DOR [37.4 (17.0–82.3)] (Fig. 6). The pooled diagnostic sensitivity (*p* = 0.808) and specificity (*p* = 0.413) between US and MRI are not significantly different in the detection of PAS.

The SROC curve for the performance of prenatal diagnosis of US and MRI for the identification of PAS are shown in Figs. 4B and 6B, respectively. Based on the SROC curve of US and MRI, we calculated the area under the curve (AUC) and Q^*^ indices for predictive accuracy and the results show that AUC and Q^*^ indices of US were 0.935 (standard error (SE), 0.017) and 0.872, that of MRI was 0.932 (SE 0.021) and 0.867, respectively. Z value statistics analysis was used to compare the AUC between US and MRI with a value of 0.1299, and there was no statistical significance with a corresponding p value of 0.5517.

### Subgroup analysis of US and MRI in the prenatal identification of PAS

Given that the predictive accuracy of imaging is evolving and can be influenced by either technique improvement or accumulative experience of sonographers and radiologists. To investigate the heterogeneity of identified 18 studies, we conducted two subgroup analyses by dividing the included studies into (1) retrospective studies versus prospective studies, (2) early studies (published before 2015) versus recent studies (published after 2015).

In the subgroup analysis of prospective studies [9, 10, 12, 17, 18, 20, 23, 24], the pooled performance of US was sensitivity 92%, specificity 86%, AUC 0.96 (SE 0.01), and that of MRI was sensitivity 93%, specificity 91%, AUC 0.96 (SE 0.02). In retrospective studies [11, 13–16, 19, 21, 22, 25, 26], the pooled performance of US was sensitivity 88%, specificity 80%, AUC 0.96 (SE 0.03) and that of MRI was sensitivity 85%, specificity 82%, AUC 0.89 (SE 0.04).

In the subgroup analysis of early studies (published before 2015) [9–17], the pooled performance of US was sensitivity 90%, specificity 88%, AUC 0.94 (SE 0.02), and that of MRI was sensitivity 90%, specificity 89%, AUC 0.97 (SE 0.01). In recent studies (published after 2015) [18–26], the pooled performance of US was sensitivity 90%, specificity 79%, AUC 0.91 (SE 0.04) and that of MRI was sensitivity 88%, specificity 85%, AUC 0.90 (SE 0.02).

There was no statistical significance (*p* > 0.05) in pooled sensitivity and specificity as well as AUC of SROC curves between US and MRI in different subgroups. The summary estimates of sensitivity, specificity, DOR, LR+, LR−, SROC curve (AUC ± SE, Q^*^ ± SE) of US and MRI for prenatal identification of PAS in the subgroup of retrospective and prospective studies, and the subgroup of earlier and recent studies are listed in Table 2, respectively.

**Table 2:** Summary estimates of sensitivity, specificity, DOR, LR+, LR−, SROC curve (AUC ± SE, Q^*^ ± SE) of US and MRI for identification of PAS in different subgroup

Subgroup		Sensitivity (%) (95% CI)	Specificity (%) (95% CI)	DOR (95% CI)	LR + (95% CI)	LR− (95% CI)	SROC curve AUC ± SE	SROC curve Q^*^ ± SE
Prosp [9, 10, 12, 17, 18, 20, 23, 24]	US	92 (87–96)	86 (81–90)	77 (27–220)	8.63 (3.21–23.16)	0.10 (0.06–0.18)	0.96 ± 0.01	0.90 ± 0.02
	MRI	93 (88–97)	91 (87–94)	96 (24–385)	8.05 (3.79–17.11)	0.12 (0.05–0.25)	0.96 ± 0.02	0.91 ± 0.03
Retro [11, 13–16, 19, 21, 22, 25, 26]	US	88 (83–92)	80 (75–85)	24 (10–57)	3.55 (2.09–6.03)	0.21 (0.13–0.34)	0.90 ± 0.03	0.83 ± 0.03
	MRI	85 (79–90)	82 (77–87)	21 (8–54)	4.09 (2.52–6.64)	0.22 (0.13–0.37)	0.89 ± 0.04	0.83 ± 0.04
Earlier studies [9–17]	US	90 (83–94)	88 (83–92)	51 (16–161)	6.24 (2.40–16.24)	0.17 (0.10–0.28)	0.94 ± 0.02	0.88 ± 0.03
	MRI	90 (84–95)	89 (85–93)	62 (13–305)	6.77 (2.68–17.11)	0.14 (0.06–0.33)	0.97 ± 0.01	0.92 ± 0.02
Recent studies [18–26]	US	90 (85–94)	79 (74–84)	32 (13–78)	4.38 (2.68–7.15)	0.16 (0.09–0.27)	0.91 ± 0.04	0.84 ± 0.04
	MRI	88 (82–92)	85 (80–88)	30 (13–67)	5.00 (3.79–6.6)	0.19 (0.11–0.32)	0.90 ± 0.02	0.83 ± 0.02

US, ultrasonography; MRI, magnetic resonance imaging; PAS, placenta accrete spectrum disorder; DOR, diagnostic odds ratio; LR+, positive likelihood ratios; LR−, negative likelihood ratios; SROC, summary receiver operating characteristic; AUC, area under the SROC curve; SE, standard error

## Discussion

Since the first study describing the ultrasound characteristics of placenta increta in the early 1980s [27], ultrasonography (US) has been used as the primary diagnostic tool for PAS. US is a widely available, inexpensive diagnostic tool and is generally considered safe for both mother and fetus. However, the diagnostic accuracy of US for identification of invasive placenta could be impaired by several clinical parameters such as unfavorable placenta site (i.e., posterior placenta) and high body mass index (BMI). Accurate prenatal diagnosis of PAS remains a challenge.

On the contrary, the imaging modality of MRI is not restricted by the depth and it offers the advantage of multiplanar imaging and excellent soft-tissue resolution. MRI demonstrates an excellent performance for defining the topography and depth of abnormal placentation [10]. MRI is often recommended when the adjacent pelvic organs involvement is highly suspected, or there is an inconclusive assessment on US, or the posterior placenta and maternal obesity affect the diagnostic accuracy of US. A recent systematic review and meta-analysis [28] was conducted to evaluate the predictive performance of MRI in detecting the severity of abnormal placental invasion and showed that the pooled sensitivity of MRI in detecting the placenta accrete was 94.4%, 100% for placenta increta, 86.5% for placenta percreta, and corresponding pooled specificity was 98.8%, 97.3%, and 96.8%, respectively. However, the included studies of the meta-analysis mainly resulted from the target population who had already been screen on US, and the MRI was used as an adjunct tool which probably contributed to a risk of selection bias. Therefore, the predictive accuracy of MRI in defining the grade of PAS might not be clearly demonstrated by these results and further more evidence is needed.

There have been more studies that assess and compare the predictive performance of US and MRI in PAS. D’Antonio et al. in a recent meta-analysis reported that the pooled sensitivity and specificity of US in diagnosing PAS were 90.7% and 96.9% [29], and subsequently reported that the pooled sensitivity and specificity of MRI in diagnosing PAS were 94.4% and 84% in another study [30]. One study comparing the diagnostic value of these two imaging tools within in one systematic review was published in 2013 by Meng et al. [31], reported that pooled sensitivity of diagnostic value for US and MRI was 83% and 82%, respectively, corresponding pooled specificity for US and MRI was 95% and 88%, respectively. Although these reviews conclude that both US and MRI were equally effective for identification of PAS, evaluation of the diagnostic performance of US and MRI were conducted on separate patient populations, which unavoidably represents a source of bias.

To precisely evaluate the predictive accuracy for PAS identification between US and MRI, we only included studies in which US and MRI were conducted within the identical patient population. In this present study, we found that the pooled sensitivity and specificity for US was 90% and 83% and that for MRI was 89% and 87%, respectively. We concluded that both of the two image modalities (US and MRI) have an acceptable diagnostic value with high sensitivity and specificity for the prenatal identification of PAS, which is in accordance with the previous reports [31]. The pooled diagnostic sensitivity (*p* = 0.808) and specificity (*p* = 0.413) between US and MRI were not significantly different in the detection of PAS. Further analysis and calculation based on SROC curves showed that AUC of US and MRI was 0.935 and 0.932, and the Q^*^ indices were 0.872 and 0.867 for US and MRI, respectively. Z value test was then used to compare the significance of the two imaging techniques for diagnosing PAS by analyzing the AUC of SROC curve, and the results revealed the Z value of 0.1299 with a corresponding p value of 0.5517, indicating that there was no statistical difference for US and MRI in predictive accuracy for PAS. To further evaluate the heterogeneity of results, we conducted subgroup analyses (i.e., retrospective vs prospective, early vs recent study) and results also showed that there was no statistical significance in sensitivity, specificity as well as AUC of SROC curves between US and MRI. All results revealed that both US and MRI possessed equal accuracy in the prenatal diagnosis of PAS, and one modality has not demonstrated superiority over the other.

Although some studies suggest that MRI may be more useful than US and expert opinion and guidelines [19, 32] recommend the use of MRI in certain scenarios (i.e., posterior placental implantation, inconclusive US assessment), more evidence is required for validation. Dwyer et al. [11] found there was no significant difference in diagnostic accuracy for prenatal identification of PAS between US and MRI, and also the performance of the two imaging modalities was not impaired by the placental site (i.e., posterior placentas). Additionally, one recent study performed by Einerson et al. [33] to evaluate the diagnostic value of MRI in the identification of PAS as a secondary tool revealed that MRI only results in 19% of correct, clinically meaningful change in the US-based PAS diagnosis, and yet an incorrect change of 17% of US-based diagnosis and a 21% of incorrect confirmation of US-based diagnosis. Furthermore, MRI offered no identifiable benefit for cases that the placenta was located in posterior or lateral position. Therefore, some researchers advocated that the employment of MRI as a secondary tool to US in prenatal identification of PAS should not be routinely recommended, and obstetric management should not be altered based on MRI findings if there is a strong indication of PAS by US with several positive features with good PPV [16, 34]. Further definite studies are required to demonstrate the diagnostic accuracy of MRI for more complete evidence.

The wide difference in diagnostic accuracy between studies can be attributed to multiple factors, including small sample size, type of study, variability of study inclusion and exclusion criteria, reference ultrasound or MRI signs of PAS, operator’s experience, equipment, gestational age and confirmative diagnosis by the gold standard modality of histopathology which is often unavailable in adherent placenta or conservatively managed cases. In particular, there has been no consensus on the reference findings on US or MRI that are indicative of PAS, and the predictive performance of these two imaging modalities is largely dependent on the experience and expertise of the sonographers and radiologists. This is further limited by the absence of a standardized training program in imaging interpretation and the relative rarity of the PAS disease.

Better sensitivity but a comparably lower specificity of either US or MRI techniques for the diagnosis of PAS, perhaps because once one feature of the diagnostic criteria was present, the diagnosis of PAS was considered, consequently the number of false positives was increased, and specificity of the test reduced. The most recent retrospective cohort study [35] exhibited that a comprehensive scoring system by combing US, MRI and clinical risk factors was superior to either modality when used alone, suggesting that a complementary approach by combing several characteristic markers from various clinical parameters into a structured and quantified system might improve the diagnostic accuracy.

There are several limitations of this study. First, we acknowledge that including the assessment of topography and grade of invasive placentas can better qualify and quantify the diagnostic efficacy of US and MRI. However, this attempt is limited by the scarcity of such information in included studies. Second, the accuracy of the meta-analysis is largely dependent on the reliability of the included research; although subgroup analysis has been performed to elucidate the heterogeneity of results, this remains as a source of unavoidable bias. Third, we did not take into account some factors such as maternal characteristics (i.e., inclusion criterion of the different studies, placental location, BMI) and gestational age for imaging that may impact the predictive performance of these two imaging modalities.

The strengths of our study include the following: (1) The comparison of diagnostic accuracy between US and MRI for the identification of PAS was examined in the identical patient population for the first time. (2) We included a larger population compared to previous publications to assess the predictive performance of US and MRI. (3) Majority of the included studies showed that depictions of both US and MRI were evaluated by a skilled and experienced sonographer or radiologist without knowledge of the pathologic results or surgical findings. (4) Both sensitivity analysis and subgroup analysis revealed the similar result that there was no statistical significance of diagnostic accuracy between US and MRI, indicating the robust outcome in the present study.

In conclusion, both US and MRI demonstrate equal efficacy in diagnosing PAS and one modality has not demonstrated superiority over the other. However, routine employment of MRI with relatively high expense in the prenatal identification of PAS should not be recommended, and further definite studies are required to demonstrate the diagnostic accuracy of MRI for more complete evidence. The development of standardized training programs and comprehensive scoring system using US, MRI and clinical risk factors can help to improve accurate prenatal diagnosis of PAS.

## Data Availability

All data generated or analyzed during this study are included in this published article.

## Abbreviations

AUC: Area under the SROC curve
DOR: Diagnostic odds ratio
LR−: Negative likelihood ratios
LR+: Positive likelihood ratios
MRI: Magnetic resonance imaging
PAS: Placenta accrete spectrum
QUADAS: Quality Assessment of Diagnostic Accuracy Studies
SROC: Summary receiver operating characteristic
US: Ultrasonography
